# TLHNMDA: Triple Layer Heterogeneous Network Based Inference for MiRNA-Disease Association Prediction

**DOI:** 10.3389/fgene.2018.00234

**Published:** 2018-07-03

**Authors:** Xing Chen, Jia Qu, Jun Yin

**Affiliations:** School of Information and Control Engineering, China University of Mining and Technology, Xuzhou, China

**Keywords:** microRNA, disease, association prediction, computational prediction model, triple layer heterogeneous network

## Abstract

In recent years, microRNAs (miRNAs) have been confirmed to be involved in many important biological processes and associated with various kinds of human complex diseases. Therefore, predicting potential associations between miRNAs and diseases with the huge number of verified heterogeneous biological datasets will provide a new perspective for disease therapy. In this article, we developed a novel computational model of Triple Layer Heterogeneous Network based inference for MiRNA-Disease Association prediction (TLHNMDA) by using the experimentally verified miRNA-disease associations, miRNA-long noncoding RNA (lncRNA) interactions, miRNA function similarity information, disease semantic similarity information and Gaussian interaction profile kernel similarity for lncRNAs into an triple layer heterogeneous network to predict new miRNA-disease associations. As a result, the AUCs of TLHNMDA are 0.8795 and 0.8795 ± 0.0010 based on leave-one-out cross validation (LOOCV) and 5-fold cross validation, respectively. Furthermore, TLHNMDA was implemented on three complex human diseases to evaluate predictive ability. As a result, 84% (kidney neoplasms), 78% (lymphoma) and 76% (prostate neoplasms) of top 50 predicted miRNAs for the three complex diseases can be verified by biological experiments. In addition, based on the HMDD v1.0 database, 98% of top 50 potential esophageal neoplasms-associated miRNAs were confirmed by experimental reports. It is expected that TLHNMDA could be a useful model to predict potential miRNA-disease associations with high prediction accuracy and stability.

## Introduction

According to the central law of molecular biology, genetic information was found to be stored in protein-coding genes (Crick et al., 1961). Recent studies have revealed that up to 70% of the human genome is transcribed into RNA, whereas protein-coding genes only make up less than 2% of total genome (Djebali et al., 2012). The majority of the human genome is made up of non-coding RNAs (ncRNAs) (Derrien et al., 2012). Based on whether transcript lengths are larger than 200 nucleotides, ncRNAs can be further divided into small ncRNA and long ncRNA (lncRNA) (Kapranov et al., 2007; Guttman et al., 2013). MicroRNAs (miRNAs) are endogenous non-coding RNAs (~22 nt) that bind to the 3′-untranslated regions (3′-UTRs) of their target RNAs (mRNAs) and control the expression of gene (Ganju et al., 2017). MiRNAs could also serve as positive regulators (Jopling et al., 2005; Vasudevan et al., 2007). Sufficient evidences indicated that thousands of miRNAs have associations with many critical biological processes (Lu et al., 2008), such as cell proliferation (Cheng et al., 2005), development (Karp and Ambros, 2005), metabolism (Alshalalfa and Alhajj, 2013), aging (Bartel, 2009), transduction (Cui et al., 2006), viral infection (Miska, 2005), and so on. Some researchers also founded that allogeneic T cell responses are regulated by miRNAs (Sun et al., 2013). It also has been shown that by attenuating shared miRNAs, competing endogenous RNAs (ceRNAs) could crosstalk and regulate each other, which is essential for regulating many biological functions (Yuan et al., 2016). Moreover, miRNA34s might be key effectors of p53 tumor-suppressor function, and their inactivation might contribute to certain cancers (Bommer et al., 2007). Recently, experiments further showed that special class of 5′-capped pre-miRNAs have been identified in both C. elegans and mouse, this promotes the understanding of the transcriptional regulation of miRNA genes themselves (Chen et al., 2017a). Therefore, it is no wonder that miRNAs are closely connected with diverse human cancer types, including breast neoplasms, lung neoplasms, colon neoplasms, kidney neoplasms, lymphoma, etc. (Pasquier and Gardès, 2016). For example, studies have implicated that miR-16-1 and miR-15a could cause chromosomal translocations in patients with chronic lymphocytic leukemia (CLL) (Calin et al., 2002). Experiments further shown that miRNAs may be a new target for the molecular targeted therapy of various cancers (Guzzi et al., 2015; Chen et al., 2017b). Thus, the identification of disease-associated miRNAs can provided a new viewpoint with the respect to the diagnosis, prevention and treatment of human complex diseases in the field of medicine (Chen, 2016). However, using the traditional biological methods to identify miRNA-disease associations is usually time-consuming and expensive. Therefore, more and more scholars have focused on developing efficient computational models to predict potential miRNA-disease associations by integrating various experimentally validated datasets. Database HMDD and miR2Disease (Jiang et al., 2009; Li et al., 2014c) have been constructed to collect the associations between human miRNAs and diseases based on previous biological experiments.

According to the assumption that functionally similar miRNAs tend to be associated with phenotypically similar diseases (Lu et al., 2008; Bandyopadhyay et al., 2010), several computational approaches have been established to infer the new miRNA-disease associations. Mork et al. (2014) introduced a computational model, named miRPD. They identified potential miRNA–disease associations by systematic combination of known miRNA-protein associations with known protein-disease associations. Shi et al. (2013) established a computational framework on the basis of the assumption that miRNAs whose target genes are associated with specific diseases are more possible to be related to these diseases. They constructed protein-protein interaction (PPI) networks and implemented random walk on the network to calculate the probability scores of each miRNA-disease pair. Xu et al. (2011) introduced an approach to infer novel human miRNA-disease associations by combining computational target prediction with expression profiles of miRNA and mRNA in tumor and nontumor tissues. In the model, the probability scores of each miRNA-disease pair could be converted into the functional similarity calculation between miRNA targets and known diseases-related genes. More importantly, the model could be a useful tool for miRNA-disease association prediction without relying on the known miRNA-disease associations. Jiang et al. (2010) proposed a computational model on the basic of hypergeometric distribution to predict new disease-associated miRNA by systematic integration of miRNA functional similarity network, disease phenotype similarity network, and experimentally verified disease-miRNA association network. However, less than 40 percent of the molecular for human disease is known and the dataset of miRNA-target interactions used in the above studies were not highly accurate, which may limit the application of the method mentioned above.

Researchers have also proposed other methods without relying on the dataset of miRNA-target interactions. For example, Chen et al. (2012b) developed the method of Random Walk with Restart for MiRNA–Disease Association (RWRMDA) to identify new disease-associated miRNAs by applying a similarity-based RWR on miRNA functional similarity network. Xuan et al. (2015) proposed the method of MIRNAs associated with Diseases Prediction (MIDP) to predict new miRNAs candidates using random walk. In which they built a miRNA network derived from miRNA-associated diseases by integration of the nodes similarities, nodes prior information and their local topological structure. Then, the potential association between a disease and a miRNA could be inferred until the iterative walking process on the network converged. Xuan et al. (2013) further proposed an effective computational approach of HDMP by comprehensive integration of miRNA functional similarity and the distribution of miRNAs associated with the disease in the *k* most similar neighbors to obtain scores of new miRNAs-disease associations. Li et al. (2017) developed Matrix Completion for MiRNA-Disease Association prediction (MCMDA), a reliable computational method in which they updated scores of each pair using matrix completion algorithm. The model is of high efficiency to update the low-rank miRNA-disease association matrix. Chen and Yan (2014) reported a method named Regularized Least Squares for MiRNA-Disease Association prediction (RLSMDA) on the basis of miRNA functional similarity, disease semantic similarity and known human miRNA-disease associations using a semi-supervised classifier. Recently, Chen et al. (2016a) introduced the model of Within and Between Score for MiRNA-Disease Association prediction (WBSMDA) by combination of integrated similarity and known miRNA-disease associations. The model built two prediction functions from the perspective of disease and miRNA according to the idea that functionally similar miRNAs tend to be associated with similar diseases, and combined them to calculate the association probability of each miRNA-disease pair. Chen et al. (2016b) further developed Heterogeneous Graph Inference for MiRNA-Disease Association prediction (HGIMDA), a new approach in which they constructed a heterogeneous network on the basic of miRNA functional similarity, disease semantic similarity, known miRNA-disease associations and an iterative update equation that propagates information across the heterogeneous network were established to infer new disease-associated miRNAs. A deep ensemble miRNA-disease association prediction (DeepMDA) framework was also introduced by Fu and Peng (2017) to identify potential miRNA-disease associations using a three-layer neural network classifier based on high-level features extracted from miRNA and disease similarity. Moreover, some other computing models for the identification of miRNA-disease associations were also gradually proposed, such as Liu et al. (2016) predicted miRNA-disease associations by implementing random walk on a heterogeneous network with multiple data sources. Zou et al. (2015) introduced two computational methods of KATZ and CATAPULT to make prediction for miRNA-disease pairs based on social network analysis methods. Pallez et al. (2017) presented a predictive approach named MiRAI using an evolutionary tuned latent semantic analysis. Pasquier and Gardès (2016) make prediction for miRNA-disease associations with a vector space model.

As mentioned above, an integration strategy may provide more comprehensive and accurate information to predict disease-related miRNAs. Actually, miRNA dysregulation is related to many human diseases through many factors, including, for example, miRNA-mRNA interactions, miRNA-lncRNA interactions, miRNA-protein interactions and so on. The miRNAs involved in genes, coding RNAs, and proteins have been used widely in other computational model for the identification of miRNA-disease associations (Shi et al., 2013) (Mork et al., 2014). In this paper, considering many experimentally verified miRNA-lncRNA interactions have been confirmed by recent biological experiments (Li et al., 2014a), we introduced the model of Triple Layer Heterogeneous Network based inference for MiRNA-Disease Association prediction (TLHNMDA) to identify the potential biological links between miRNAs and diseases by integrating multi-level data regarding miRNAs, diseases, lncRNAs and their association information into a triple layer heterogeneous network. We implemented leave-one out cross validation (LOOCV) and 5-fold cross validation on the TLHNMDA to evaluate its performance. The AUCs of LOOCV were respectively 0.8795, and the model obtained the average AUC of 0.8795 ± 0.0010 on 5-fold cross validation. Then, case studies of kidney neoplasms, prostate neoplasms and lymphoma were implemented to assess the independent prediction performance of the model. As a result, 42, 38, and 39 out of top 50 potential miRNAs for these three important diseases were confirmed in dbDEMC (Yang et al., 2010) and miR2Disease (Jiang et al., 2009) database, respectively. We further tested TLHNMDA on the database HMDD v1.0 (Lu et al., 2008) to see whether the TLHNMDA still performs well. Taking esophageal neoplasms as an example, as a result, 49 of the top 50 esophageal neoplasms-associated miRNAs were verified by experimental reports. It has proved that TLHNMDA is reliable and effective in predicting potential disease-associated miRNAs.

## Materials and methods

### Human miRNA-disease association

In this paper, the known dataset of human miRNA–disease associations were downloaded from HMDD v2.0 database. The dataset contains 383 diseases, 495 miRNAs and 5430 high-quality experimentally verified human miRNA-diseases associations. Furthermore, an adjacency matrix *A* was established to denote known miRNAs-disease associations. The row of the matrix represents the disease, and the column represents the miRNAs. We used the variables *nm* and *nd* to represent the number of miRNAs and diseases in the dataset, respectively. The value of *A(d(i), m(j))* is 1 when miRNA *m(i)* is associated with disease *d(j)*, otherwise 0.

### miRNA-lncRNA interactions

The dataset of miRNA-lncRNA interactions can be obtained from starBase v2.0 database (Li et al., 2014a), which provided the most comprehensive experimentally confirmed miRNA–lncRNA interactions. The dataset consists of 10112 known miRNA-lncRNA interactions about 132 miRNAs and 1114 lncRNAs. In addition, the known lncRNAs-related miRNAs that do not appear in the dataset of known miRNA-disease associations mentioned above is deleted. As a result, 9088 miRNA-lncRNA interactions were obtained. We also constructed an adjacency matrix *B* to represent known miRNA-lncRNA interactions. The row of the *B* represents the miRNAs, and the column represents the lncRNAs. The variable *nl* represents the number of lncRNA in the dataset. If miRNA *m(i)* is interacted with lncRNA *l(j)*, the value of *B(m(i), l(j))* in the *B* is 1, otherwise 0.

### miRNA functional similarity

Wang et al. (2010) introduced a computational method of miRNA functional similarity between a miRNA pair (*m*_*i*_ and *m*_*j*_). The whole process of the computational method can be divided into four steps. First, we need to identify the diseases set *D*(*m*_*i*_) (diseases related to *m*_*i*_) and *D*(*m*_*j*_) (diseases related to *m*_*j*_) for miRNA *m*_*i*_ and *m*_*j*_, respectively. Second, in both sets, the semantic values of all diseases are calculated according to the corresponding DAG. Third, the semantic similarity for each disease pairs between *D*(*m*_*i*_) and *D*(*m*_*j*_) can be computed by consideration of their semantic value. In the last step, the functional similarity between *m*_*i*_ and *m*_*j*_ is calculated in the light of the semantic similarity obtained in step three. From http://www.cuilab.cn/files/images/cuilab/misim.zip, miRNA functional similarity probability scores can be downloaded. Similarly, we built matrix *FS* to stand for the miRNA functional similarity matrix, where *FS(m(i), m(j))* is the functional similarity probability score between miRNA *m(i)* and *m(j)*.

### Disease semantic similarity model 1

Each disease can be described as a Directed Acyclic Graph (DAG). For example, disease *D* can be denoted as *DAG(D)* = *(D,T(D),E(D))*, where *T(D)* is a set of node *D* itself and its ancestor nodes, *E(D)* stands for the edges between parent and child nodes (Wang et al., 2010). Therefore, the semantic value of disease *D* could be calculated as follows:

(1) $$DV1\left(D\right)=\sum_{d\in T\left(D\right)}D_{D}1\left(d\right)$$

where

(2) $$\{\begin{array}{cc} D_{D}1(d)=1 & if\;d=D\\ D_{D}1(d)=\max\{\Delta^{*}D_{D}1(d^{\prime })\; & |d^{\prime }\in children\;of\;d\}\;if\;d\neq D \end{array}\;$$

Δ is the semantic contribution factor. For disease *D*, the contribution of itself to the semantic value of disease *D* is 1. If the distance between *D* and *d* increases, the semantic contribution value of disease *d* to the D will decreases. Thus, if diseases in the same layer, they would have the same contribution to the semantic value of disease *D*. The value of semantic similarity in disease semantic similarity model 1 between disease *d(i)* and *d(j)* can be defined as follows:

(3) $$SS1\left(d\left(i\right),d\left(j\right)\right)=\frac{\sum_{t\in T(d(i))\cap T(d(j))}\left(D_{d\left(i\right)}1\left(t\right)+(D_{d\left(j\right)}1\left(t\right)\right)}{DV1\left(d\left(i\right)\right)+DV1(d(j))}$$

### Disease semantic similarity model 2

In the disease semantic similarity model 2, considering different disease terms in the same layer of DAG(*D*) may appear in different numbers of disease DAGs, disease with more specific which appears in less disease DAGs should contribute to the semantic similarity of disease *D* at a higher contribution level. Therefore, the contribution of disease *d* to the semantic value of disease *D* can be calculated as follows:

(4) $$D_{D}2\left(d\right)=-log[\frac{the\;number\;of\;DAGs\;including\;d}{the\;number\;of\;diseases}]$$

In disease semantic similarity model 2, the value of semantic similarity between *d(i)* and *d(j)* can be defined as follows:

(5) $$\text{SS2}\left(d\left(i\right),d\left(j\right)\right)=\frac{\sum_{t\in T(d(i))\cap T(d(j))}\left(D_{d\left(i\right)}2\left(t\right)+(D_{d\left(j\right)}2\left(t\right)\right)}{DV2\left(d\left(i\right)\right)+DV2(d(j))}$$

### Gaussian interaction profile kernel similarity

Gaussian interaction profile kernel similarity for diseases can be defined based on the known miRNA-disease associations dataset by considering the assumption that similar diseases tend to be related with more common miRNAs. In this paper, the binary vector *IP(d(u))* is the *u*th row of matrix A, which was used to indicate the interaction profiles between disease *d(u)* and each miRNA. Therefore, the value of Gaussian interaction profile kernel similarity between diseases *d(u)* and *d(v)* is defined as follows.

(6) $$KD\left(d\left(u\right),d\left(v\right)\right)=\exp(-\gamma_{d}||IP\left(d\left(u\right)\right)-IP\left(d\left(v\right)\right)|{|}^{2})$$

where parameter γ_*d*_ is used to control the kernel bandwidth, which can be obtained from the normalization of a new bandwidth ${\gamma^{\prime }}_{d}$ by the average number of associated miRNAs for all the diseases.

(7) $$\gamma_{d}=\frac{{\gamma^{\prime }}_{d}}{(\frac{1}{nd}\sum_{n=1}^{nd}\left||IP(d(u))|{|}^{2}\right)}$$

Similarly, we defined the value of Gaussian interaction profile kernel similarity between miRNA *m(i)* and *m(j)* as follows:

(8) $$KM\left(m\left(i\right),m\left(j\right)\right)=\exp(-\gamma_{m}||IP\left(m\left(i\right)\right)-IP\left(m\left(j\right)\right)|{|}^{2})$$

(9) $$\gamma_{m}=\frac{{\gamma^{\prime }}_{m}}{(\frac{1}{nm}\sum_{n=1}^{nm}\left||IP(m(i))|{|}^{2}\right)}$$

Gaussian interaction profile kernel similarity for lncRNA *l(i)* and *l(j)* can also be calculated as follows:

(10) $$KL\left(l\left(i\right),l\left(j\right)\right)=\text{exp}(-\gamma_{l}||IP\left(l\left(i\right)\right)-IP\left(l\left(j\right)\right)|{|}^{2})$$

(11) $$\gamma_{l}=\frac{{\gamma^{\prime }}_{l}}{(\frac{1}{nl}\sum_{n=1}^{nl}\left||IP(l(i))|{|}^{2}\right)}$$

(12) $$SM\left(m\left(i\right)\text{​},m\left(j\right)\right)=\{\begin{array}{ll} \frac{KM\left(m\left(i\right),m\left(j\right)\right)+FS(m\left(i\right),m(j))}{2}\; & m\left(i\right)\;and\;m\left(j\right)\;has\;functional\;similarity\;\\ KM\left(m\left(i\right),m\left(j\right)\right)\; & otherwise \end{array}\;\text{​​​​​​​​}$$

(13) $$SD\left(d\left(i\right)\text{​},d\left(v\right)\right)=\{\begin{array}{ll} \frac{KD\left(d\left(u\right),d\left(v\right)\right)+SS(d\left(u\right),d(v))}{2}\; & d\left(u\right)\;and\;d\left(v\right)\;has\;similarity\\ KD\left(d\left(u\right),d\left(v\right)\right)\; & otherwise \end{array}\text{​​​​​​​​}$$

### Integrated similarity for miRNAs and diseases

Here, integrated miRNA similarity matrix *SM* are defined on the basis of miRNA functional similarity and Gaussian interaction profile kernel similarity for miRNAs. Integrated disease similarity matrix *SD* are constructed according to disease semantic similarity and Gaussian interaction profile kernel similarity for diseases.

where

(14) $$SS=\frac{SS1+SS2}{2}$$

### TLHMDA

**Figure 1:**
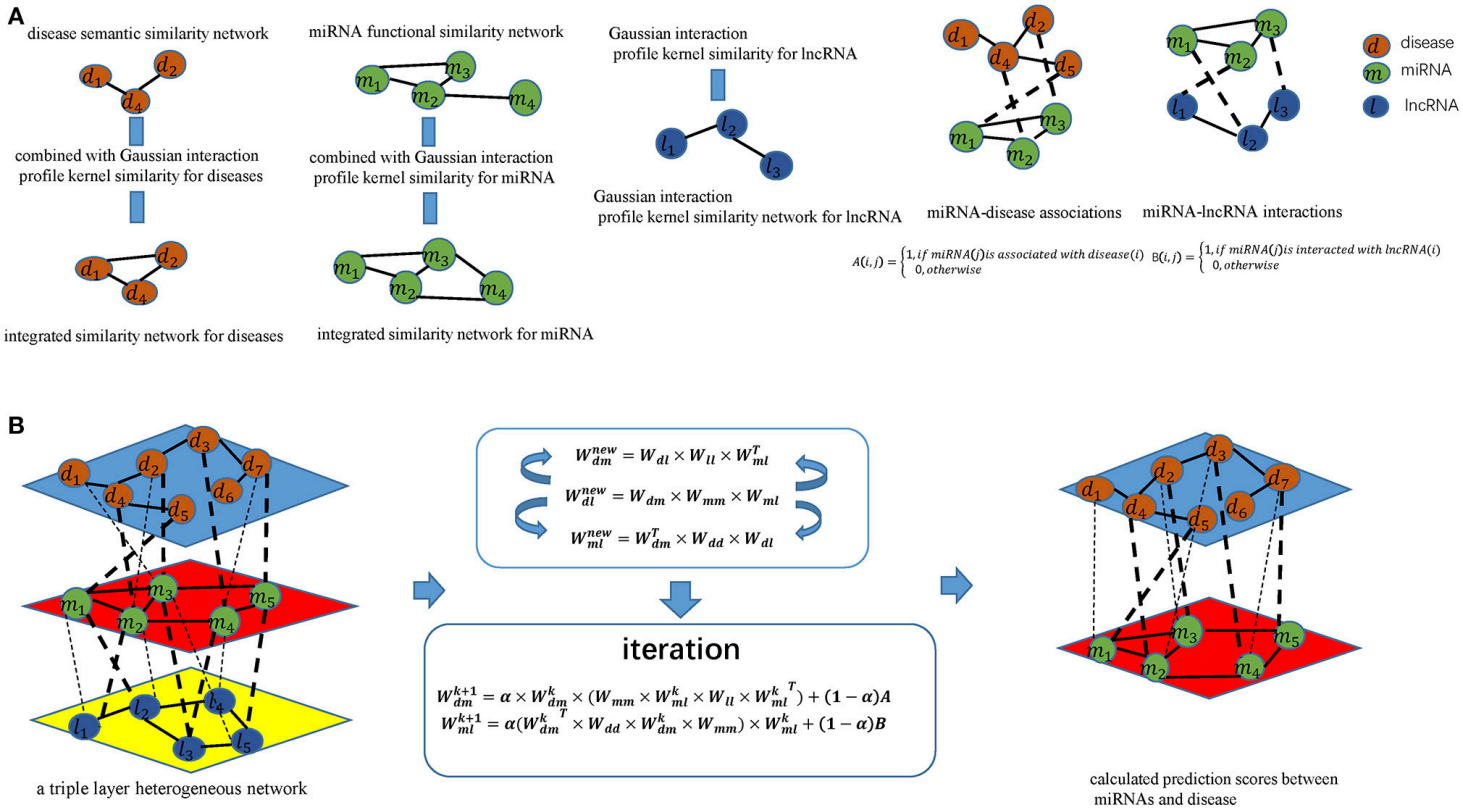
Flowchart of potential disease-miRNA association prediction based on the computational model of TLHNMDA: **(A)** Constructing miRNA-disease association matrices, miRNA-lncRNA interaction matrices and obtaining integrated similarity network by combining miRNA functional similarity, disease semantic similarity, Gaussian interaction profile kernel similarity; **(B)** Constructing a triple layer heterogeneous network and predicting potential miRNA-disease associations based on an iterative equation to obtain the stable association probability.

According to the guilt-by-association principle (Barabási et al., 2011), new miRNA–disease associations can be inferred through existing associations between similar miRNAs and similar diseases, Likewise, novel miRNA-lncRNA interactions can be inferred through existing associations between similar miRNA and lncRNA (see Figure 1). We infer new miRNA-lncRNA associations in the newly proposed triple layer heterogeneous network by using an information flow-based method. New disease-lncRNA association matrix $W_{dl}^{new}$ could be constructed as follows:

(15) $$W_{dl}^{new}=W_{dm}\times SM\times W_{ml}$$

As shown in the above formula, we can identify potential disease-lncRNA associations on the basis of miRNA-disease associations *W*_*dm*_, miRNA-lncRNA interactions *W*_*ml*_ as well as integrated similarity for miRNAs *SM* according to the equation. Once the associations between diseases and lncRNAs are established. New association $W_{dm}^{new}$ between diseases and miRNAs can be defined by considering these associations:

(16) $$W_{dm}^{new}=W_{dl}\times KL\times W_{ml}^{T}$$

Equation (16) is potentially more powerful in capturing miRNA-disease associations by incorporating lncRNA information into miRNA-disease prediction. As a by-product from the model, we can also obtain a new interaction between each miRNA and lncRNA pair by incorporating miRNA-disease associations *W*_*dm*_, disease-lncRNA associations *W*_*dl*_ as well as integrated similarity for miRNAs *SD*. New association $W_{ml}^{new}\;$between miRNAs and lncRNAs can be defined as follows:

(17) $$W_{ml}^{new}=W_{dm}^{T}\times SD\times W_{dl}$$

where the superscript T indicates the transpose of the corresponding matrix.

We treat *W*_*dl*_ as a temporary value, and replace *W*_*dl*_ in the two Equations (16, 17) using the Equation (15), respectively.

(18) $$W_{dm}^{new}=W_{dm}\times SM\times W_{ml}\times KL\times W_{ml}^{T}$$

(19) $$W_{ml}^{new}=W_{dm}^{T}\times SD\times W_{dm}\times SM\times W_{ml}$$

Once the new miRNA-disease associations $W_{dm}^{new}$ and new miRNA-lncRNA interactions $W_{ml}^{new}$were obtained, we established iterative updating procedure based on Equations (18, 19). The final computational model can be written as follows:

(20) $$W_{dm}^{k+1}=\alpha \times W_{dm}^{k}\times \left(SM\times W_{ml}^{k}\times KL\times {W_{ml}^{k}}^{T}\right)+\left(1-\alpha \right)A$$

(21) $$W_{ml}^{k+1}=\alpha \left({W_{dm}^{k}}^{T}\times SD\times W_{dm}^{k}\times SM\right)\times W_{ml}^{k}+\left(1-\alpha \right)B$$

Here α a decay factor in the range of (0,1). *A* and *B* represents the initial disease–miRNA associations and miRNA–lncRNA interactions, respectively. $W_{dm}^{k}$and $W_{ml}^{k}$ would be converge with proper normalization utilizing Equations (24, 25), respectively (Wang et al., 2013c) (the proof can be found in the Supplementary Materials).

(22) $$IM=SM\times W_{ml}^{k}\times KL\times {W_{ml}^{k}}^{T}$$

(23) $$ID={W_{dm}^{k}}^{T}\times SD\times W_{dm}^{k}\times SM$$

(24) $$\begin{array}{l} IM(m(i),m(j))=\\ \frac{IM(m(i),m(j))}{\sqrt{\sum_{l=1}^{nm}IM(m(i),m(l))}\sqrt{\sum_{l=1}^{nm}IM(m(j),m(l))}} \end{array}$$

(25) $$\begin{array}{l} ID(d(i),d(j))=\\ \frac{ID(d(i),d(j))}{\sqrt{\sum_{l=1}^{nd}ID(d(i),d(l))}\sqrt{\sum_{l=1}^{nd}ID(d(j),d(l))}} \end{array}$$

After some steps, the iteration will be stable after some steps (the change in value between $W_{dm}^{k+1}$ and $W_{dm}^{k}$ measured by L1 norm is less than a given cutoff, the cutoff in this paper was 10^−6^).

The three-layer model is proposed by incorporating miRNA-lncRNA information into miRNA-disease association prediction based on miRNA dysregulation is associated with many human complex diseases may through miRNA-lncRNA interactions. It can be seen from the two iterative algorithms, once new association between miRNA and disease is estimated, it can be used to update other miRNA-disease associations and miRNA-lncRNA interactions. Similarly, once new association between miRNA and lncRNA is estimated, it can also be used to update other miRNA-disease associations and miRNA-lncRNA interactions. Therefore, the layer between miRNA and disease and the layer between miRNA and lncRNA paly the same important role in the triple layer heterogeneous network to propagate information for the identification of potential miRNA-disease associations and miRNA-lncRNA interactions simultaneously. In order to make the two constructed iterative equations to work effectively, known miRNA-disease associations and known miRNA-lncRNA interactions as weights were added to the inferred equations because the initial links deserve more credibility. At last, $W_{dm}^{new}$and $W_{ml}^{new}$ were expected to converge, which means that the propagation of information would be stable at the end.

## Results

### Performance evaluation

**Figure 2:**
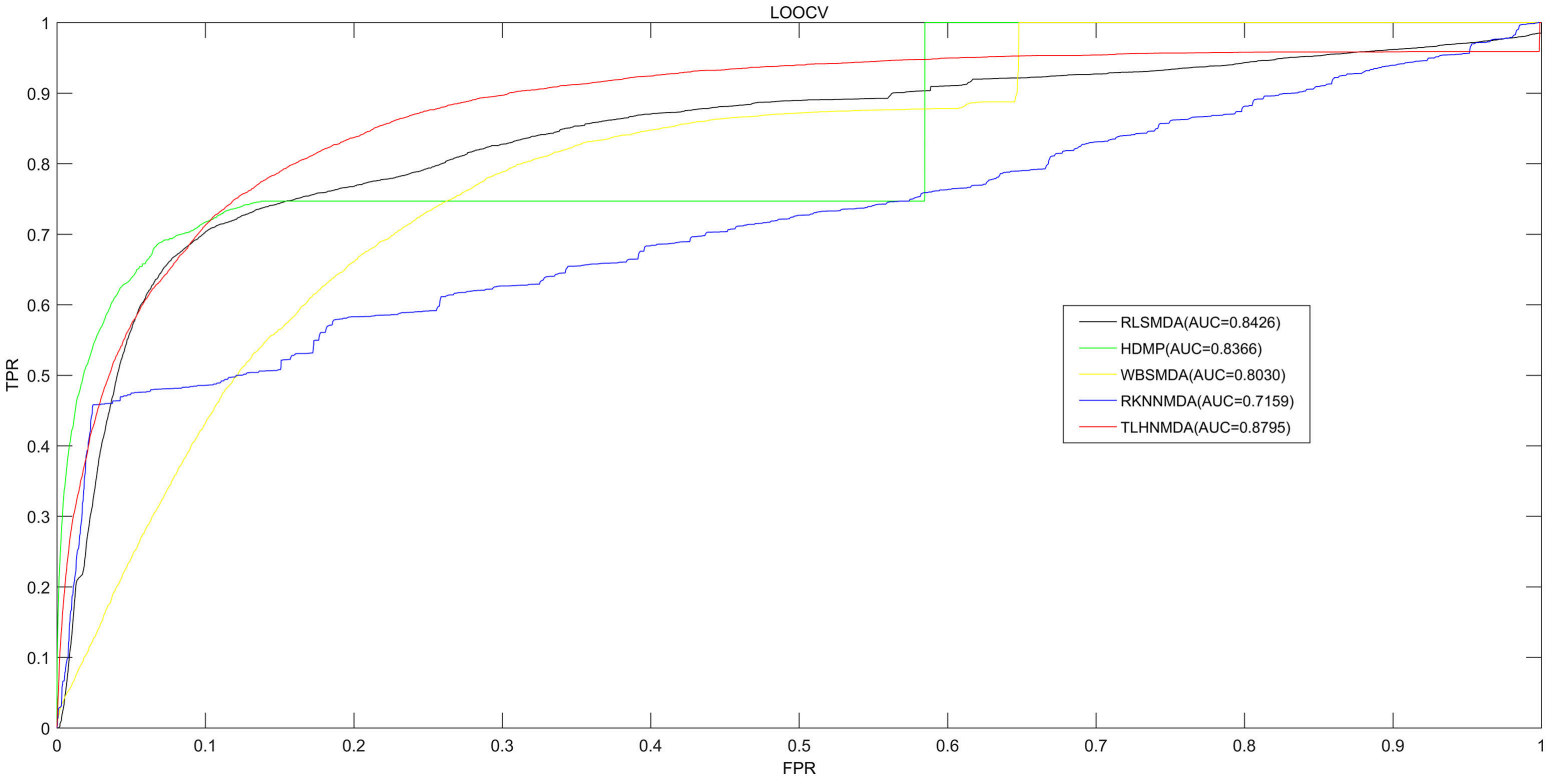
Comparison between TLHNMDA, RLSMDA, HDMP, WBSMDA, RKNNMDA in terms of ROC curve and AUC based on LOOCV. As a result, TLHNMDA, RLSMDA, HDMP, WBSMDA, RKNNMDA achieved AUCs of 0.8795, 0.8426, 0.8366, 0.8030, and 0.7159 in the LOOCV, respectively. In conclusion, TLHNMDA outperform the other models.

We implemented LOOCV as well as 5-fold cross validation on the basis of the experimentally verified miRNA-disease associations in HMDD v2.0 database (Li et al., 2014c) to evaluate the prediction performance of TLHNMDA. Moreover, TLHNMDA were compared with four previous classical computational methods: RLSMDA (Chen et al., 2012b), HDMP (Xuan et al., 2013), WBSMDA (Xu et al., 2011), RKNNMDA (Chen et al., 2017c). In the framework of LOOCV evaluation, each known association of miRNA-disease pair in the database was considered as test samples in turn, the other known miRNA-disease associations were considered as training samples, the miRNA-disease pairs with no known verified associations were regarded as candidate samples. After TLHNMDA was implemented, we would obtain the scores of the test samples and the scores of the candidate samples, and then the score of the test sample was compared with the scores of all the candidate samples in LOOCV. While in 5-fold cross validation, the experimentally verified miRNA-disease associations were evenly divided into five disjoint parts. One part was selected as test samples and the other four parts were regarded as training samples in each time. Similarly, the miRNA-disease pairs without known association evidences were regarded as candidate samples. Then, the score of each test sample was compared with the scores of all the candidate samples. It is worth noting that the above process was repeated 100 times, we would get 100 rankings for all miRNA and disease pairs. It is worth noting that almost all the models for the prediction of miRNA-disease associations according to the assumption that miRNAs with similar functions tend to be related to phenotypically similar diseases were proposed based on the LOOCV and 5-fold cross validation (Mork et al., 2014; Xuan et al., 2015; You et al., 2017; Zhong et al., 2017). At last, we drew Receiver Operating Characteristics (ROC) curve using true positive rate (TPR, sensitivity) against the false positive rate (FPR, 1-specificity) at different thresholds evaluate the performance of TLHNMDA clearly. Sensitivity refers to the percentage of the positive miRNA-disease associations whose score ranks are higher than the preset threshold, while specificity refers to the percentage of negative miRNA-disease pairs with ranks lower than the threshold. Then, the value of Area under the ROC curve (AUC) could be calculated to evaluate the prediction performance of the model. If the value of AUC is 1, it tells us the approach possesses perfect prediction performance; if the value of AUC is 0.5, it stands for the method possesses random prediction performance. For LOOCV, TLHNMDA, RLSMDA, HDMP, WBSMDA, RKNNMDA obtained AUCs of 0.8795, 0.8426, 0.8366, 0.8030 and 0.7159, respectively (see Figure 2). For 5-fold, TLHNMDA, RLSMDA, HDMP, WBSMDA, RKNNMDA obtained the average AUCs and corresponding standard deviations of 0.8795 ± 0.0010, 0.8569 ± 0.0020, 0.8342 ± 0.0010, 0.8185 ± 0.0009, and 0.6723 ± 0.0027, respectively.

### Case studies

Here, to evaluate the prediction accuracy of TLHNMDA, case studies were implemented on kidney neoplasms, lymphoma and prostate neoplasms. In the model, the 5430 known miRNA-disease associations in HMDD v2.0 were utilized as the training set. All candidate miRNAs for each interested disease were ranked in accordance with their predicted scores. After that, the top 50 predicted miRNAs were picked out and verified in other two important miRNA-disease association databases (i.e., dbDEMC and miR2Disease). Furthermore, the results showed that 232 of the 5430 known miRNA-disease associations in HMDD v2.0 also existed in miR2Disease and 546 known associations also existed in dbDEMC. It is noteworthy that there was no overlap between the training samples and the prediction lists. That is because only candidate miRNAs (miRNAs have any no known associations with interested disease in HMDD v2.0) for interested disease were ranked and verified in case studies. Accordingly, none of the top 50 predicted miRNAs existed in HMDD v2.0 and the verification of miRNAs in the prediction lists was completely independent of HMDD v2.0.

Kidney neoplasms, known as renal cancer, is a common health problem in cancer diseases (Manojlovi et al., 1986). The age of its incidence can be in all ages, particularly in the age between 50 and 70 years old (Nickerson et al., 2002). The most common symptoms of kidney neoplasms patients are pains in the lumbar and hematuria (Duque et al., 1998). Many existing treatments of kidney neoplasms are usually radiation therapy and chemotherapy drugs, which do not have much effect in the cure (Zbar et al., 2003). Up to now, lots of miRNAs have been reported to be associated with kidney neoplasms. For example, miRNA-192, miRNA-194, miRNA-215, miRNA-200c, and miRNA-141 were proved to be associated with renal childhood neoplasms (Senanayake et al., 2012). MiRNA-210 was reported to be upregulated in renal neoplasms (Eilertsen et al., 2014). Another miRNA named miRNA-23b could act as an oncogene and reducing the expression of miRNA-23b would be an effective way to inhibit the growth of kidney tumor, which might contribute to the treatment of renal neoplasms in medicine (Liu et al., 2010). In case studies, we implemented TLHNMDA on kidney neoplasms to predict the potential miRNA-disease associations. In short, 8 of the top 10 and 42 of the top 50 novel identified miRNAs associated with kidney neoplasms were validated by the two database deDEMC and miR2Disease (see Table 1).

**Table 1:** Prediction of the top 50 predicted miRNAs associated with kidney neoplasms based on known associations in HMDD v2.0 database.

**miRNA**	**Evidence**	**miRNA**	**Evidence**
hsa-mir-16	dbDEMC	hsa-mir-20a	dbDEMC miR2Disease
hsa-mir-15b	dbDEMC	hsa-mir-539	unconfirmed
hsa-mir-195	dbDEMC	hsa-mir-26a	dbDEMC miR2Disease
hsa-mir-424	dbDEMC miR2Disease	hsa-mir-27b	dbDEMC
hsa-mir-497	dbDEMC	hsa-mir-34a	dbDEMC
hsa-mir-103a	unconfirmed	hsa-mir-17	miR2Disease
hsa-mir-485	unconfirmed	hsa-mir-29b	dbDEMC miR2Disease
hsa-mir-23a	dbDEMC	hsa-mir-125b	unconfirmed
hsa-mir-214	dbDEMC miR2Disease	hsa-mir-143	dbDEMC
hsa-mir-155	dbDEMC	hsa-mir-128	dbDEMC
hsa-mir-107	dbDEMC	hsa-mir-320a	unconfirmed
hsa-mir-590	unconfirmed	hsa-mir-708	unconfirmed
hsa-mir-19a	dbDEMC	hsa-mir-124	dbDEMC
hsa-mir-125a	dbDEMC	hsa-mir-149	dbDEMC
hsa-mir-142	unconfirmed	hsa-mir-199a	dbDEMC miR2Disease
hsa-mir-19b	dbDEMC miR2Disease	hsa-mir-34c	dbDEMC
hsa-mir-138	dbDEMC	hsa-mir-181a	dbDEMC
hsa-mir-26b	dbDEMC	hsa-mir-152	dbDEMC
hsa-mir-150	dbDEMC miR2Disease	hsa-mir-106a	dbDEMC miR2Disease
hsa-mir-29c	dbDEMC miR2Disease	hsa-mir-18a	dbDEMC
hsa-mir-370	dbDEMC	hsa-mir-181b	dbDEMC
hsa-mir-31	dbDEMC	hsa-mir-193a	dbDEMC
hsa-mir-185	dbDEMC miR2Disease	hsa-mir-7	dbDEMC miR2Disease
hsa-mir-24	dbDEMC	hsa-mir-122	dbDEMC miR2Disease
hsa-mir-29a	dbDEMC miR2Disease	hsa-mir-106b	dbDEMC miR2Disease

*The first column records top 1–25 related miRNAs. The second column records the top 26–50 related miRNAs*.

Lymphoma is the fastest growing human tumor (Chen et al., 2013), which is a group of blood cell tumors develop from lymphocytes (a type of white blood cell). The disease consists of two categories: Hodgkin lymphomas (HL) and the non-Hodgkin lymphomas(NHL) (Mcduffie et al., 2009). Many lymphoma-related miRNAs have been reported based on recent biological experiments. For example, the expression of miRNA-150 was confirmed to be a tumor suppressor in malignant lymphoma (Watanabe et al., 2011), which induces the differentiation of EBV-positive Burkitt lymphoma differentiation based on the modulation of c-Mybi *in vitro* (Li et al., 2014a). In addition, miR-21 could regulate cell activity of proliferation, invasion, and apoptosis. Accordingly, it has a potential therapeutic application in lymphoma (Sekar et al., 2014). We implemented TLHNMDA on lymphoma to predict the top 10 and top 50 related miRNAs. Briefly speaking, 7 of top 10 and 39 of top 50 potential lymphoma-related miRNAs were verified in the deDEMC and miR2Disease database (see Table 2).

**Table 2:** Prediction of the top 50 predicted miRNAs associated with lymphoma based on known associations in HMDD v2.0 database.

**miRNA**	**Evidence**	**miRNA**	**Evidence**
hsa-mir-15b	dbDEMC	hsa-mir-199a	dbDEMC
hsa-mir-195	dbDEMC	hsa-mir-34c	unconfirmed
hsa-mir-424	dbDEMC	hsa-mir-152	dbDEMC
hsa-mir-497	dbDEMC	hsa-mir-106a	dbDEMC miR2Disease
hsa-mir-103a	unconfirmed	hsa-mir-181b	dbDEMC
hsa-mir-485	unconfirmed	hsa-mir-193a	unconfirmed
hsa-mir-23a	dbDEMC	hsa-mir-7	dbDEMC
hsa-mir-214	dbDEMC	hsa-mir-106b	dbDEMC
hsa-mir-107	dbDEMC	hsa-mir-22	dbDEMC
hsa-mir-590	unconfirmed	hsa-mir-27a	dbDEMC
hsa-mir-142	unconfirmed	hsa-mir-144	unconfirmed
hsa-mir-26b	dbDEMC	hsa-mir-326	dbDEMC
hsa-mir-370	unconfirmed	hsa-mir-93	dbDEMC
hsa-mir-31	dbDEMC	hsa-mir-186	dbDEMC
hsa-mir-185	dbDEMC	hsa-mir-30a	dbDEMC
hsa-mir-23b	dbDEMC	hsa-mir-148a	dbDEMC
hsa-mir-29a	dbDEMC	hsa-mir-182	dbDEMC
hsa-mir-27b	dbDEMC	hsa-mir-199b	dbDEMC
hsa-mir-34a	dbDEMC	hsa-mir-145	dbDEMC miR2Disease
hsa-mir-29b	dbDEMC	hsa-mir-328	dbDEMC miR2Disease
hsa-mir-125b	unconfirmed	hsa-mir-330	dbDEMC
hsa-mir-143	dbDEMC miR2Disease	hsa-mir-421	unconfirmed
hsa-mir-128	dbDEMC	hsa-mir-1	dbDEMC
hsa-mir-320a	unconfirmed	hsa-mir-181c	dbDEMC
hsa-mir-149	dbDEMC miR2Disease	hsa-mir-141	dbDEMC

*The first column records top 1–25 related miRNAs. The second column records the top 26–50 related miRNAs*.

Prostate neoplasms is the most common disease in men (Siegel et al., 2013). The malignant tumor originates from prostate in the epithelial cells (Gmyrek et al., 2001). In the recent years, many miRNAs have been verified to be related with prostate neoplasms base on accumulating researches. For instance, miR-141, miR-375, miR-21, miR-93, miR-106a, miR-874, miR-1207, and miR-26a were reported to upregulate in prostate neoplasms (Xiao et al., 2012; Chu et al., 2014; Dong et al., 2015). We also implemented TLHNMDA on prostate neoplasms to identify the related miRNAs. As a result, 7 of top 10 and 38 of top 50 potential Prostate neoplasms-miRNAs were confirmed in the deDEMC and miR2Disease database (see Table 3).

**Table 3:** Prediction of the top 50 predicted miRNAs associated with prostate neoplasms based on known associations in HMDD v2.0 database.

**miRNA**	**Evidence**	**miRNA**	**Evidence**
hsa-mir-15a	dbDEMC miR2Disease	hsa-mir-24	dbDEMC miR2Disease
hsa-mir-16	dbDEMC miR2Disease	hsa-mir-29a	dbDEMC miR2Disease
hsa-mir-15b	dbDEMC	hsa-mir-539	unconfirmed
hsa-mir-195	dbDEMC miR2Disease	hsa-mir-20a	miR2Disease
hsa-mir-424	unconfirmed	hsa-mir-26a	dbDEMC miR2Disease
hsa-mir-497	miR2Disease	hsa-mir-34a	dbDEMC miR2Disease
hsa-mir-103a	unconfirmed	hsa-mir-27b	dbDEMC miR2Disease
hsa-mir-485	unconfirmed	hsa-mir-29b	dbDEMC miR2Disease
hsa-mir-23a	dbDEMC miR2Disease	hsa-mir-17	miR2Disease
hsa-mir-214	dbDEMC miR2Disease	hsa-mir-143	dbDEMC miR2Disease
hsa-mir-155	dbDEMC	hsa-mir-128	dbDEMC
hsa-mir-107	unconfirmed	hsa-mir-320a	unconfirmed
hsa-mir-590	unconfirmed	hsa-mir-708	unconfirmed
hsa-mir-19a	dbDEMC	hsa-mir-124	dbDEMC
hsa-mir-125a	dbDEMC miR2Disease	hsa-mir-149	dbDEMC miR2Disease
hsa-mir-142	unconfirmed	hsa-mir-199a	dbDEMC miR2Disease
hsa-mir-19b	dbDEMC miR2Disease	hsa-mir-34c	dbDEMC
hsa-mir-138	dbDEMC	hsa-mir-181a	dbDEMC miR2Disease
hsa-mir-26b	dbDEMC miR2Disease	hsa-mir-152	dbDEMC
hsa-mir-150	dbDEMC	hsa-mir-18a	unconfirmed
hsa-mir-370	miR2Disease	hsa-mir-21	dbDEMC miR2Disease
hsa-mir-29c	dbDEMC	hsa-mir-106a	dbDEMC miR2Disease
hsa-mir-31	dbDEMC miR2Disease	hsa-mir-181b	dbDEMC miR2Disease
hsa-mir-185	unconfirmed	hsa-mir-193a	unconfirmed
hsa-mir-23b	dbDEMC miR2Disease	hsa-mir-7	dbDEMC

*The first column records top 1–25 related miRNAs. The second column records the top 26–50 related miRNAs*.

Moreover, we further implemented TLHNMDA on the known miRNA-disease associations in HMDD v1.0 database (Lu et al., 2008) to see whether the approach worked properly on a different dataset. Consequently, the predicted scores for candidate miRNAs showed that 10 of top 10 and 49 of top 50 potential esophageal neoplasms-associated miRNAs were verified by three databases (see Table 4). Lastly, we list the potential miRNAs related to all the human diseases and the association scores of the entire ranking results obtained by the computational model of TLHNMDA (see Supplementary Table 1).

**Table 4:** Prediction of the top 50 predicted miRNAs associated with esophageal neoplasms based on HMDD v1.0 database.

**miRNA**	**Evidence**	**miRNA**	**Evidence**
hsa-mir-15a	dbDEMC and HMDD	hsa-mir-143	dbDEMC and HMDD
hsa-mir-16	dbDEMC	hsa-mir-29a	dbDEMC
hsa-mir-15b	dbDEMC	hsa-mir-125b	dbDEMC
hsa-mir-195	dbDEMC	hsa-mir-29b	dbDEMC
hsa-mir-424	dbDEMC	hsa-mir-181b	dbDEMC
hsa-mir-497	dbDEMC	hsa-mir-34a	dbDEMC HMDD
hsa-mir-214	dbDEMC HMDD	hsa-mir-106a	dbDEMC
hsa-mir-107	dbDEMC miR2Disease	hsa-mir-106b	dbDEMC
hsa-mir-155	dbDEMC HMDD	hsa-mir-199a	dbDEMC HMDD
hsa-mir-19a	dbDEMC HMDD	hsa-mir-330	dbDEMC
hsa-mir-19b	dbDEMC	hsa-mir-20b	dbDEMC
hsa-mir-125a	dbDEMC	hsa-mir-26a	dbDEMC HMDD
hsa-mir-185	dbDEMC	hsa-mir-1	dbDEMC
hsa-mir-20a	dbDEMC HMDD	hsa-mir-181a	dbDEMC
hsa-mir-24	dbDEMC	hsa-mir-186	dbDEMC
hsa-mir-17	dbDEMC	hsa-mir-141	dbDEMC HMDD
hsa-mir-23a	dbDEMC	hsa-mir-93	dbDEMC
hsa-mir-26b	dbDEMC	hsa-mir-421	dbDEMC
hsa-mir-539	unconfirmed	hsa-mir-222	dbDEMC
hsa-mir-150	dbDEMC HMDD	hsa-mir-28	dbDEMC HMDD
hsa-mir-23b	dbDEMC	hsa-mir-145	dbDEMC HMDD
hsa-mir-29c	dbDEMC HMDD	hsa-mir-92a	HMDD
hsa-mir-370	dbDEMC	hsa-mir-22	dbDEMC HMDD
hsa-mir-142	dbDEMC	hsa-mir-199b	dbDEMC
hsa-mir-18a	dbDEMC	hsa-mir-34c	dbDEMC HMDD

*The first column records top 1–25 related miRNAs. The second column records the top 26–50 related miRNAs*.

## Discussion

Although progress has been made in the discovery of miRNA, the role of miRNAs in physiologic and pathophysiologic processes is just emerging. MiRNAs as governors of gene expression during cardiovascular development and disease have associations with many critical biological processes (Liu and Olson, 2010). Identification of miRNAs expressed in specific cardiac cell types may provide us with new diagnostic, prognostic, and therapeutic targets for many forms of cardiovascular disease (Cordes and Srivastava, 2009). Furthermore, aberrant expression of miRNAs has also been involved in various neurological disorders (NDs) of the central nervous system such as alzheimer disease, parkinson's disease, huntington disease, amyotrophic lateral sclerosis, schizophrenia and autism. If dysregulated miRNAs are found in patients with NDs, this may also be a biomarker for the earlier diagnosis and monitoring of disease progression. Identifying the role of miRNAs in normal cellular processes is critical in the development of new therapeutic strategies for NDs (Kamal et al., 2015). Therefore, predicting disease-associated miRNAs is important for the understanding of disease pathogenesis and treatment of a variety of clinically important disease. In this paper, according to the hypothesis that functional similar miRNAs and lncRNAs are likely to be associated with similar diseases. We introduced a novel model, named TLHNMDA, which constructed a triple layer heterogeneous network by systematic combination of miRNA functional similarity, disease semantic similarity, Gaussian interaction profile kernel similarity, known miRNA-disease associations and miRNA-lncRNA interactions to identify new disease-associated miRNAs. In the model, an iterative updating algorithm that propagates information across the network was proposed based on the triple layer heterogeneous graph to obtain final prediction scores between diseases and miRNAs. The experimental results from LOOCV and 5-fold cross validation have demonstrated that TLHNMDA outperforms other four computational methods. What's more, case studies of four human diseases: kidney neoplasms, lymphoma, prostate neoplasms and esophageal neoplasms were implemented and the results were verified by the experimental literatures in dbDEMC and miR2Disease database. We can see that the TLHNMDA turns out to be more reliable and effective in inferring the potential miRNA–disease associations than the previous computational models. Therefore, our model could be an effective and useful computational model to predict new miRNA-disease associations. Biomedical researchers could use TLHNMDA to computationally identify the miRNAs that were potentially related to the investigated diseases.

TLHNMDA could obtain the valid performances due to the following several reasons. Firstly, TLHNMDA improved prediction accuracy and decrease the prediction bias by integration of several reliable types of biological datasets, including the accurate experimentally verified miRNA-disease associations, known miRNA-lncRNA interactions, miRNA functional similarity network, disease semantic similarity network and Gaussian interaction profile kernel similarity. Secondly, the model captured new miRNAs-diseases associations using global network similarity information, it has an advantage over the local network similarity information model to capture miRNA-disease associations. Finally, TLHNMDA is an iterative algorithm to update predicted scores based on global network similarity information until the state is in convergence, which promote the effective prediction of TLHNMDA. However, several limitations also exist in the TLHNMDA, for example, TLHNMDA cannot predict the new miRNAs associated with the new diseases without any known miRNA-disease associations. Besides, there is no powerful methods to find optimal parameters of TLHNMDA. The selection of parameters in the iterative algorithm is based on past experiences which can't guarantee the model with best state in the implementation process. Finally, the number of miRNA-disease associations and miRNA-lncRNA interactions, confirmed by biological experiments, is still insufficient. Therefore, in the future research, we can have a try to propose a new model by integrating more available biological datasets.

It is noteworthy that there exist many other types of data can also be used to predict miRNA-disease associations, for example, miRNA-mRNA interactions (Li et al., 2014b), miRNA-protein interactions (Shi et al., 2016), miRNA-environmental factors interactions (Chen et al., 2012a), and so on. Considering some existing methods have taken advantage of different datasets to identify miRNA-disease associations, which makes direct comparison of their performance and the performance of the proposed method is not realistic. For example, two model proposed by Pallez et al. (2017) and Pasquier and Gardès (2016) were based on the dataset of miRNA-disease associations, miRNA-neighbor associations, miRNA-target associations, miRNA-word associations and miRNA-family associations. The model proposed by Mork et al. (2014) was based on the dataset of miRNA–protein associations and protein-disease associations to predict potential miRNA-disease associations. The model introduced by Shi et al. (2013) for the identification of miRNA-disease associations was based on disease-gene association, protein-protein interaction, miRNA-target associations. Moreover, Liu et al. (2016) proposed a new computational to predict unobserved miRNA-disease associations based on disease functional similarity, disease semantic similarity and miRNA similarity. It is worth noting that miRNA similarity in the model was calculated based on miRNA-lncRNA interactions. In addition to datasets, there are different ways in defining relationships among nodes of the same type. For example, in the DeepMDA proposed by Fu and Peng (2017), Gaussian interaction profile kernel similarity for disease was calculated by using three association matrices, the miRNA-disease association matrix, the lncRNA-disease association matrix, and the gene-disease association matrix. MiRNA similarity used in KATZ and CATAPULT introduced by Zou et al. (2015) was calculated by text mining analysis of their phenotype descriptions in the Online Mendelian Inheritance in Man (OMIM) database. Especially, the relative merits of using different measures are worth further study. Network analysis and modeling researches constructed by diverse data were also widely applied in other fields. Some studies modeled cancer cells by constructing and modeling networks for individual clones based on tumor genome sequencing (Wang et al., 2013a). Integrative network modeling has been applied in the modeling of drug resistance for personalized treatment (Wang et al., 2013b). Moreover, Hallmark-specific networks were modeled to better understand key cellular processes, which are involved in cancer development and progression (Gao et al., 2016). The hallmarks of cancer are one of the most widely acknowledged organizing principles for research on cancer (Wang et al., 2015). Accumulating evidences indicated that there are some associations between cancer hallmarks and genes (Wang et al., 2015). For example, miR-16 obtained the highest score in the case study on kidney neoplasms and the second high score in the case study on prostate neoplasms. APP, ATG12, and ATF2 are the common targets for this miRNA and have been identified to be involved in hallmark of inflammation (Wang et al., 2015). In the future work, we plan to extend the model we proposed into new multi-layer prediction model, one extension is to add more diverse datasets of different types (other than the three discussed here) and more associations to the model, then construct the iterative updating algorithm to identify disease-associated miRNAs.

## Author contributions

XC conceived the project, developed the prediction method, designed the experiments, analyzed the result, and wrote the paper. JQ implemented the experiments, analyzed the result, and wrote the paper. YJ analyzed the result and revised the paper.

### Conflict of interest statement

The authors declare that the research was conducted in the absence of any commercial or financial relationships that could be construed as a potential conflict of interest.
