# Supplementary material for: TLHNMDA: Triple Layer Heterogeneous Network Based Inference for MiRNA-Disease Association Prediction
Source: Front Genet. 2018 Jul 3;9:234. doi: 10.3389/fgene.2018.00234 (PMC6038677; doi:10.3389/fgene.2018.00234)
Supplement: Supplementary file 2 [file Data_Sheet_2.docx]

**TLHNMDA: Triple Layer Heterogeneous Network based inference for MiRNA-Disease Association prediction**

**Xing Chen^1,*^, Jia Qu^1^, Jun Yin^1^**

^1^School of Information and Control Engineering, China University of Mining and Technology, Xuzhou, 221116, China

*** Correspondence:**Xing Chen

[xingchen@amss.ac.cn](mailto:xingchen@amss.ac.cn)

**Keywords: microRNA, disease, association prediction, computational prediction model, triple layer heterogeneous network**

1. $W_{dm}^{k}$and $W_{ml}^{k}$ defined in equations 20 and 21 in the main text will converge after normalization defined in the Proof.

**Proof:**

For a matrix $A={(a_{i,j})}_{n\times m}$ define function *normalize(),* which takes matrix *A* as input and apply the following transformation to all its elements:$a_{i,j}=\frac{a_{i,j}}{\sqrt{\sum_{k=1}^{m} a_{i,k}}\sqrt{\sum_{k=1}^{n} a_{k,j}}}$.

Let

$f(W_{ml})=normalize(SM\times W_{ml}\times KL\times W_{ml}^{T})$

$f(W_{dm})=normalize({W_{dm}^{k}}^{T}\times SD\times W_{dm}^{k}\times SM)$

Then equation 20 and 21 can be rewritten as:

$$W_{dm}^{k+1}=\alpha W_{dm}^{k}\times f(W_{ml}^{k})+\left( 1-\alpha\right)A$$

$W_{ml}^{k+1}=\alpha f\left( W_{dm}^{k} \right)\times W_{ml}^{k}+\left( 1-\alpha\right)B$

Suppose the transformation step used in the proof of (Wang, et al., 2013) as$G$.

$$W_{dm}^{{k+1}^{*}}=\alpha{G(I,f(W_{ml}^{k}))W}_{dm}^{k^{*}}+\left( 1-\alpha\right)A^{*}$$

$W_{ml}^{{k+1}^{*}}=\alpha G(f\left( W_{dm}^{k} \right),I)W_{ml}^{k^{*}}+\left( 1-\alpha\right)B^{*}$

$\left[ {W_{dm}^{{k+1}^{*}} \atop W_{ml}^{{k+1}^{*}}} \right] =\alpha\left[ \begin{matrix} G\left( I,f\left( W_{ml}^{k} \right) \right), & 0 \\ 0, & G(f\left( W_{dm}^{k} \right),I) \end{matrix} \right]\left[ \begin{matrix} W_{dm}^{k^{*}} \\ W_{ml}^{k^{*}} \end{matrix} \right]$+$\left( 1-\alpha\right)\left[ \begin{matrix} A^{*} \\ B^{*} \end{matrix} \right]$

The eigenvalue of $\left[ \begin{matrix} G\left( I,f\left( W_{ml}^{k} \right) \right), & 0 \\ 0, & G(f\left( W_{dm}^{k} \right),I) \end{matrix} \right]$ is in the range of $[-1,1]$. Therefore, we have$\left\| \begin{matrix} W_{dm}^{k^{*}} \\ W_{ml}^{k^{*}} \end{matrix} \right\|\leq\left\| \begin{matrix} A^{*} \\ B^{*} \end{matrix} \right\|$

We must prove that $H(\left[ \begin{matrix} X_{1} \\ X_{2} \end{matrix} \right]) =\alpha\left[ \begin{matrix} G\left( I,f\left( X_{2} \right) \right), & 0 \\ 0, & G(f\left( X_{1} \right),I) \end{matrix} \right]\left[ \begin{matrix} X_{1} \\ X_{2} \end{matrix} \right]$+$\left( 1-\alpha\right)\left[ \begin{matrix} X_{1}^{0} \\ X_{2}^{0} \end{matrix} \right]$ is a contraction mapping on the domain$\left\| X \right\|\leq\left\| X^{0} \right\|$. Apparently, for any given *X,* we have $\left\| H(X) \right\|\leq\left\| X^{0} \right\|$. Because $\left\| G\left( I,f\left( X \right) \right) \right\|$ is continuous, for any pair of vector $\left[ \begin{matrix} X_{11} \\ X_{12} \end{matrix} \right]$ and $\left[ \begin{matrix} X_{21} \\ X_{22} \end{matrix} \right]$ in the domain $\left\| X \right\|\leq\left\| X^{0} \right\|$, there is a $\lambda>0$.

$$\left\| \left[ \begin{matrix} G\left( I,f\left( X_{12} \right) \right)-G\left( I,f\left( X_{22} \right) \right), & 0 \\ 0, & G\left( f\left( X_{11} \right),I \right)-G\left( f\left( X_{21} \right),I \right) \end{matrix} \right] \right\|<\lambda\left\| \left[ \begin{matrix} X_{11} \\ X_{12} \end{matrix} \right]-\left[ \begin{matrix} X_{21} \\ X_{22} \end{matrix} \right] \right\|$$

$$\left\| H(\left[ \begin{matrix} X_{11} \\ X_{12} \end{matrix} \right])-H(\left[ \begin{matrix} X_{21} \\ X_{22} \end{matrix} \right]) \right\|=\alpha\left\| \left[ \begin{matrix} G\left( I,f\left( X_{12} \right) \right), & 0 \\ 0, & G(f\left( X_{11} \right),I) \end{matrix} \right]\left[ \begin{matrix} X_{11} \\ X_{12} \end{matrix} \right]-\left[ \begin{matrix} G\left( I,f\left( X_{22} \right) \right), & 0 \\ 0, & G(f\left( X_{21} \right),I) \end{matrix} \right]\left[ \begin{matrix} X_{21} \\ X_{22} \end{matrix} \right] \right\|$$

$\mathbf{=}\alpha\left\| \left[ \begin{matrix} G\left( I,f\left( X_{12} \right) \right), & 0 \\ 0, & G(f\left( X_{11} \right),I) \end{matrix} \right]\left[ \begin{matrix} X_{11}-X_{21} \\ X_{12}-X_{22} \end{matrix} \right]+ \left[ \begin{matrix} G\left( I,f\left( X_{12} \right) \right)-G\left( I,f\left( X_{22} \right) \right), & 0 \\ 0, & G\left( f\left( X_{11} \right),I \right)-G(f\left( X_{21} \right),I) \end{matrix} \right]\left[ \begin{matrix} X_{21} \\ X_{22} \end{matrix} \right] \right\|< \alpha\left\| \left[ \begin{matrix} X_{11}-X_{21} \\ X_{12}-X_{22} \end{matrix} \right] \right\|$+$\alpha\lambda\left\| \left[ \begin{matrix} X_{11}-X_{21} \\ X_{12}-X_{22} \end{matrix} \right] \right\|\left\| X^{0} \right\|$

=$\alpha(1+\lambda\left\| X^{0} \right\|)\left\| \left[ \begin{matrix} X_{11}-X_{21} \\ X_{12}-X_{22} \end{matrix} \right] \right\|$

Once we have $\left\| X^{0} \right\|$ small enough, we can have

$\left\| H(\left[ \begin{matrix} X_{11} \\ X_{12} \end{matrix} \right])-H(\left[ \begin{matrix} X_{21} \\ X_{22} \end{matrix} \right]) \right\|<\left\| \left[ \begin{matrix} X_{11}-X_{21} \\ X_{12}-X_{22} \end{matrix} \right] \right\|$. Therefore *H* is a contraction mapping and there is a fixed point for *H* in $\left\| (X) \right\|\leq\left\| X^{0} \right\|$. In fact, in our calculation, we have *H* converged without any normalization on $X^{0}$.

# Supplementary Table

**Supplementary Table 1.** We applied TLHNMDA to prioritize all the candidate miRNA-disease pairs based on all the known miRNA-disease associations in HMDD database as training samples. This prediction result is released for further experimental validation and research.
